# Characterization of patients with anti-modified citrullinated vimentin antibodies (MCVA)

**DOI:** 10.1186/1479-5876-9-S2-P52

**Published:** 2011-11-23

**Authors:** Estíbaliz Ruiz-Ortiz de Arrizabaleta, Dolors Grados-Cánovas, Aina Teniente-Serra, Iñaki Salvador-Corres, Ana Marín, Melania Martínez-Morillo, Susana Holgado, Alejandro Olivé, Ricardo Pujol-Borrell, Eva Martínez-Cáceres

**Affiliations:** 1 Immunology Laboratory, Banc de Sang I Teixits, Hospital Universitari Germans Trias i Pujol (HUGTP), Badalona, Spain; 2 Dept. of Cell Biology, Physiology and Immunology, Faculty of Medicine, Universitat Autònoma Barcelona, Spain; 3 Rheumatology Division, HUGTP, Badalona, Spain; 4 Immunology Division, Hospital Universitari Vall d’Hebron, Barcelona, Spain

## Introduction

Measurament of MCVA is used for the diagnosis of rheumatoid arthritis (RA), with similar sensitivity to but higher specificity than rheumatoid factor. MCVA, have been detected however in other diseases and also in some healthy subjects.

## Aim

To analyze the clinical features of patients positive for anti-MCVA.

## Patients and methods

Retrospective analysis of MCVA+ve patients detected over a period of 3 years in a reference laboratory. 335 patients (32.5% men and 67.5% women) with anti-MCVA ≥20U/ml fulfilled inclusion criteria.

## Results

241/335 (71.9%) MCVA+ve patients had a rheumatic condition; most (67%) of RA patients had values >100U/ml and corresponded to seropositive cases with erosions. Among vasculitis and connective tissue disease patients, values were in the 20-60U/ml range. Only 7 of them, 4 lupus, two SS and one polyarteritis nodosa, had values >60U/ml. Distribution in Still's disease, palindromic rheumatism and elderly-onset arthritis was similar. 73% of non-filiated arthritis were 20-60U/ml. In the miscellaneous group, psoriatic and microcrystalline arthritis, polymyalgia rheumatica, sarcoidosis, arthritis associated to inflammatory bowel disease and postinfectious arthritis, 71.4% were <60U/ml. Only one case of polymyalgia rheumatica and one of chondrocalcinosis was >100U/ml.

94/335 cases (28.1%) had no rheumatic disease. Of them the six cases with MCVA >100U/ml were two nephropaties, two lung diseases, one autoimmune hemolytic anemia and one drepanocytosis.

## Conclusions

Although anti-MCVA are considered highly specific for RA, they are also detected in other rheumatic diseases and in non rheumatic disorders. Values >60U/ml give a higher positive predictive value (PPV) for RA diagnosis, while a cut-off at >20U/ml, reduces PPV markedly. Therefore, in patients with no articular symptoms, values in the range 20-60U/ml should be considered with caution.

